# Psychometric Properties of the Persian Self‐Advocacy in Cancer Survivorship Scale (SACS‐P) in Iran

**DOI:** 10.1002/cam4.71318

**Published:** 2025-10-29

**Authors:** Elaheh Habibpour, Nargess Ramazanzadeh, Sevda Gardashkhani, Mehraban Shahmari

**Affiliations:** ^1^ Department of Medical‐Surgical Nursing, School of Nursing and Midwifery Ardabil University of Medical Sciences Ardabil Iran; ^2^ Student Research Committee, Tabriz University of Medical Sciences Tabriz Iran

**Keywords:** advocacy, cancer survivors, psychometric properties, self‐advocacy

## Abstract

**Background:**

Self‐advocacy is crucial for cancer patients, as it allows them to express their needs and engage in treatment decisions. This skill enhances the quality of care and life satisfaction. Accurately measuring self‐advocacy and supporting patients with lower skills is essential to improving treatment outcomes and promoting patient‐centered care. Accurate measurement of self‐advocacy is vital for identifying patients with lower skills and providing support to enhance treatment outcomes and patient‐centered care. This study aimed to investigate the psychometric properties of the Persian version of the Self‐Advocacy Scale in Cancer Patients (SACS).

**Methods:**

This methodological study used a cross‐sectional design with 339 cancer patients selected through convenience sampling. It examined the scale's face, content, construct validity, and reliability after translation and cultural adaptation.

**Results:**

The original English version of the Self‐Advocacy in Cancer Survivorship Scale (SACS) consisted of 29 items, five of which were removed based on content validity results. This reduction resulted in the Persian version (SACS‐P) containing 24 items. These items were grouped into three dimensions: informed decision‐making, effective communication with healthcare providers, and connected strength. The scale showed satisfactory validity, with exploratory factor analysis (EFA) revealing three latent factors that accounted for 52.85% of the total variance. Confirmatory factor analysis (CFA) confirmed the adequacy of the three‐factor model. The overall Cronbach's alpha was 0.89, and the intraclass correlation coefficient (ICC) was 0.75 with a 95% confidence interval. Convergent validity was supported by a moderate correlation (*r* = 0.49) with the Persian version of the Patient Self‐Advocacy Scale for chronic illnesses (PSAS‐P).

**Conclusions:**

The SACS‐P (24‐item) is a reliable and valid instrument for assessing self‐advocacy in cancer patients in Iran, suitable for use in both research and clinical settings.

## 1 Introduction

Cancer remains a critical global health challenge, ranking among the leading causes of mortality worldwide [1]. According to statistics published in 2020, 19.3 million new cases were reported, with half occurring in the Asian population [2]. In Iran, the burden is significant, with an estimated 115,000 new cases in 2020, projected to rise by 42.6% to approximately 160,400 new cases by 2025 [3]. The confluence of population growth, demographic aging, and increased exposure to carcinogenic agents has precipitated a surge in cancer incidence, particularly within developing nations [1, 4]. Recent advancements in cancer treatment have improved survival rates, leading to a growing number of cancer survivors. Consequently, with the increasing number of cancer patients globally—resulting from higher incidence rates and lower mortality rates—practical care for individuals affected by cancer has emerged as a critical issue in healthcare [4, 5].

Cancer profoundly impacts patients beyond physical health, imposing significant socioeconomic challenges including disruptions in employment, financial instability, and reduced quality of life [6]. These multifaceted difficulties complicate patients' ability to engage effectively with healthcare systems and make informed decisions about their treatment. Consequently, patients face barriers in communicating their needs and accessing essential support from family and healthcare providers [7, 8].

To address these challenges, healthcare providers are encouraged to adopt a patient‐centered approach that emphasizes patient involvement in treatment decisions. This approach is grounded in three core principles: the expertise of healthcare professionals, the responsiveness of healthcare organizations, and critically, the active and informed participation of patients throughout their treatment journey [9].

The contemporary trend of prioritizing patient‐centered healthcare and encouraging greater patient participation in cancer management has significantly increased the focus on improving patients' ability to advocate for themselves [10]. In this context, the emphasis on patient self‐advocacy has acquired significant importance. Self‐advocacy emerges as a crucial skill empowering patients to navigate these challenges. Self‐advocacy enables individuals to articulate their needs and preferences, make informed decisions, effectively communicate with healthcare teams, and balance support from social networks [11, 12]. It comprises three interrelated skills: the patient's capacity to make informed decisions regarding their health, the ability to communicate effectively with the healthcare team, and the skill to balance providing and receiving support from friends and family [11, 12, 13].

Improving self‐advocacy among cancer patients has significant benefits: it enhances adherence to treatment, increases satisfaction with healthcare interactions, and fosters ethical, patient‐centered care [11]. Disease and pain are better managed in these patients, resulting in an enhanced quality of life. Furthermore, self‐advocacy supports the principle of autonomy (including key principles of medical ethics) and promotes patient‐centered care within clinical settings [11, 13].

While the notion of self‐advocacy has been thoroughly documented within groups of patients experiencing diverse health challenges, including those with HIV/AIDS, individuals with disabilities, and those facing mental health conditions [14, 15, 16], its recognition among cancer patients is limited [11] Specifically, it was in 2021 that Thomas et al. developed the Female Self‐Advocacy in Cancer Survivorship Scale (FSACS), designed to evaluate self‐advocacy behaviors specifically among women diagnosed with cancer. However, a notable constraint of this evaluative measure was its exclusion of male patients and the self‐advocacy actions unique to them. Consequently, Thomas and their team innovated the Self‐Advocacy Scale (SACS), which broadened the scope of assessment to include the self‐advocacy practices of both male and female patients [17, 18].

In the context of Iran, where cultural and linguistic factors may influence the expression and understanding of self‐advocacy, it is imperative to have a localized measurement tool [17]. Existing instruments, such as the Persian version of the Patient Self‐Advocacy Scale (PSAS‐P), are designed for chronic diseases and may not fully capture the cancer‐specific experiences of patients [19, 20]. Implementing assessment instruments not expressly developed for oncology populations may lead to a deficit in comprehensive understanding and nuanced insight regarding patient circumstances among researchers and healthcare practitioners. Additionally, the potential for inadequate evaluation of self‐advocacy competencies through non‐specialized instruments poses a risk of overlooking patients exhibiting diminished self‐advocacy capabilities, consequently impeding healthcare professionals' provision of targeted supportive interventions [17, 20].

Therefore, establishing a psychometrically robust instrument, characterized by both validity and reliability, for the explicit assessment of self‐advocacy within the cancer patient demographic is imperative to ensure the delivery of optimal and efficacious care [17]. Our research identified a gap in the translation and psychometric evaluation of the SACS into Persian. Therefore, this study aimed to investigate the psychometric properties of the SACS‐P, enhancing support for cancer patients and improving patient‐centered care in Iran.

## 2 Methods

### 2.1 Design and Setting

This study is a methodological investigation that was conducted as a cross‐sectional study in Ardabil's oncology department to validate the Persian version of the scale developed by Thomas et al. [18]. The research was carried out in two distinct phases: the first phase involved translating the scale into Persian, adhering to the guidelines set forth by the World Health Organization (WHO). In the second phase, various methods were applied, including assessments of face validity, content validity, construct validity, and internal consistency and stability evaluations to measure the scale's reliability.

#### 2.1.1 Participants

The population under investigation in this study consisted of all cancer patients who sought treatment in the oncology department of Ardabil city. The participant count was set to be 10 times the number of items in the SACS scale [21]; since the original instrument comprises 29 items and accounts for a potential 10% dropout rate, the minimum required sample size was determined to be at least 320 individuals. This 10% dropout adjustment is supported by Chow et al. (2007), who note that dropout rates of 10%–20% are standard in clinical studies [22]. It should be noted that the sample size may differ at various stages of the study, as indicated in the descriptions of each phase. The sampling method utilized in this study was convenient and accessible. The inclusion criteria included patients who were 18 years or older, had a definitive cancer diagnosis, were aware and knowledgeable about their diagnosis, and provided consent to participate in the study. Exclusion criteria included patients with severe cognitive impairments, diagnosed psychiatric disorders, or those undergoing treatment with medications known to significantly affect cognitive functions (e.g., sedatives or antipsychotics). These conditions could impede their ability to accurately complete the questionnaire. Additionally, participants who did not fully complete the questionnaire were excluded from the final analysis.

#### 2.1.2 Data Collection

Before initiating the research, all necessary permissions were obtained, and coordination was established with the relevant officials. The sampling for this study occurred from January 2025 to April 2025. The following instruments were employed for data collection:

#### 2.1.3 Demographic Information Form

This form contained questions to assess the participants' age, gender, marital status, employment status, level of education, type of cancer, health insurance, and income.

#### 2.1.4 Self‐Advocacy in Cancer Survivorship Scale (SACS)

Thomas et al. (2024) investigated the psychometric properties of the 20‐item Female Self‐Advocacy in Cancer Survivorship scale (FSACS) and added 10 new items to assess self‐advocacy in male cancer patients. During the validation process of this instrument, to determine whether the factors identified in the original scale remained valid in this sample, Exploratory Factor Analysis (EFA) was conducted using Parallel Analysis with Principal Axis Factoring. Subsequently, the researchers employed oblique rotation to determine the optimal placement of each item within the factors. Notably, the two‐factor solution (80.60% variance explained) was examined, but the three‐factor solution was preferred due to its higher explained variance of 87.94%. Additionally, item number 29 was removed at this stage due to its low correlation with other items. In the subsequent Confirmatory Factor Analysis (CFA) stage, the three‐factor solution was also deemed more appropriate than the two‐factor solution (combining the first and second factors) and was found to be more consistent with the original instrument. Consequently, the SACS scale, comprising 29 items across three factors—Informed Decision‐Making (11 items), Effective Communication with Healthcare Providers (9 items), and Connected Strength (9 items)—was established. This scale is capable of measuring self‐advocacy in both men and women. The original scale reported a Cronbach's alpha coefficient of 0.84 for the entire instrument, with subscale coefficients of 0.86, 0.65, and 0.83, respectively. Participants responded to each item using a 6‐point Likert scale, ranging from 1 (strongly disagree) to 6 (strongly agree). Higher scores on each factor and the total score indicate greater self‐advocacy. The maximum scores for the factors are 66, 54, and 54, respectively, with a total maximum score of 174 [18].

#### 2.1.5 Patient Self‐Advocacy Scale‐Persian Version (PSAS‐P)

The Persian version of the PSAS consists of 12 items, categorized into three dimensions: Increased illness education (4 items), increased assertiveness (4 items), and the potential for mindful nonadherence (4 items). Responses are gathered using a 5‐point Likert scale, ranging from 1 (strongly agree) to 5 (strongly disagree). This scale measures self‐advocacy among chronic patients aged 25–75 years. The instrument's face validity, content validity, and reliability were assessed in 2015 within the chronic patient population in Iran. It should be noted that the quantitative face validity and content validity indices for all 12 items surpassed the acceptable threshold. During the translation and validation process, only minor adjustments were made, and no items were removed; furthermore, the intra‐class correlation coefficient (ICC) for the items was estimated to be between 0.1 and 0.8 with a 95% confidence interval. The Cronbach's alpha coefficients for the instrument's dimensions were reported as 0.70, 0.60, and 0.62, with an overall instrument score of 0.75.

### 2.2 Assessment of Psychometric Properties

#### 2.2.1 Phase One: Translation Process

To evaluate the psychometric properties, the process began with translating the scale. This translation adhered to the guidelines set by the WHO [23]. Three people well‐versed in the research concept and proficient in Persian and English independently translated the original questionnaire into Persian. Afterward, they collectively selected the most appropriate equivalent for each item, leading to a unified Persian version. Discrepancies between translators were resolved through consensus during these discussions. Next, two bilingual individuals, unaware of the original scale and research stages, back‐translated the Persian version into English. The research team then compared and reviewed this back‐translated version with the original scale. Furthermore, this back‐translated version was sent to Dr. Teresa Hagan Thomas, the original designer, for approval, resulting in the final Persian questionnaire.

#### 2.2.2 Phase Two: Instrument Validation

In the validation phase, it is crucial to assess the validity of new instruments before they are used in research, as the reliability of results depends on the instrument's validity [24]. This ensures that the instrument measures what it is supposed to. Following the Consensus‐based Standards for the selection of health Measurement Instruments (COSMIN) checklist [25] the validation of the SACS scale involved three methods to evaluate its validity—face, content, and construct validity—and two methods to assess reliability—internal consistency and test–retest reliability.

##### 2.2.2.1 Face Validity

A combination of quantitative and qualitative methods was utilized to assess the face validity of the scale. Initially, 15 cancer patients were requested to evaluate the clarity of each item on a 5‐point Likert scale. Following this, the score for each item was computed using the impact formula:

Impact score (IS) = frequency (%) × comprehensiveness, where Comprehensiveness refers to the importance or relevance of each item as rated by participants [26]. According to the results obtained from this calculation, items that received a score exceeding 1.5 were deemed suitable; nonetheless, items with lower scores were not eliminated but were revised during the qualitative face validity phase based on participant feedback [27]. In the subsequent step, 15 members of the target study population were interviewed face‐to‐face and invited to share their perspectives on each item's difficulty, appropriateness, or ambiguity [28]. The research team evaluated the insights provided by the participants, leading to the implementation of necessary adjustments.

##### 2.2.2.2 Content Validity

A combination of quantitative and qualitative methodologies was applied to evaluate content validity. In the quantitative aspect, a panel of 10 experts was purposively selected, including one oncology specialist, two oncology nurses, and seven experienced faculty members with expertise in cancer research and psychometrics. These experts were invited to review the questionnaire for grammatical correctness, the suitability of word choices, the arrangement of items, scoring techniques, and other pertinent factors. They were asked to submit their feedback in writing to the researcher. Afterward, the concerns raised by the experts were addressed to better satisfy the requirements of the target population.

In the quantitative phase, content validity was assessed through two methods: the content validity ratio (CVR) and the content validity index (CVI) [21]. For the CVR assessment, specialists were asked to evaluate the necessity of each item within the tool using a 3‐point Likert scale that included options for essential, helpful but not essential, and not essential. The researcher then computed the CVR for each item according to the following formula:

$$\mathrm{CVR}=\left(n_{e}-\left(N/2\right)\right)/\left(N/2\right)$$

The CVR can range from −1 to 1, where higher values indicate a more substantial agreement among experts regarding the necessity of the item in question. Based on Lawshe's table, since the opinions of 10 specialists were utilized at this stage, a CVR value above 0.62 is considered acceptable [29]. In this research, items with a CVR score exceeding 0.62 were considered statistically significant and necessary (*p* < 0.05). Furthermore, for the CVI assessment, 10 specialists were asked to evaluate the items based on their relevance to the construct being measured using a 4‐point Likert scale (with options: 1: not relevant, 2: somewhat relevant, 3: acceptable relevance, and 4: highly relevant). The CVI for each item (I‐CVI) and the overall instrument (S‐CVI) was then calculated. The I‐CVI was determined by dividing the number of specialists rated the item as 3 or 4 by the total number of evaluating specialists. The S‐CVI was obtained by averaging the I‐CVI values. In this study, values exceeding 0.79 were deemed appropriate [30], It is important to note that in the current study, an S‐CVI greater than 0.9 was considered suitable [21].

##### 2.2.2.3 Construct Validity

To assess the construct validity of the Persian version of the SACS scale for cancer patients in Iran, the study utilized EFA to determine the latent factors. The sample size was set at 10 times the number of scale items, requiring a minimum of 320 participants. Before conducting EFA and CFA, the univariate and multivariate distributions of the data were assessed to verify normality assumptions critical for robust factor extraction and model fitting. Univariate normality was evaluated using the one‐sample Kolmogorov–Smirnov (K‐S) test on the total SACS score (*N* = 339) and supplemented by skewness and kurtosis computations for each of the 24 retained items. Critical ratios (c.r.) were derived to test significance, with a threshold of |c.r.| > 1.96 for *p* < 0.05. Multivariate normality was examined via Mardia's coefficient, encompassing multivariate skewness (β_1_) and kurtosis (β_2_) with associated c.r. values. These tests were conducted in SPSS (v.24) for K‐S and AMOS (v.24) for skewness/kurtosis and Mardia's analyses. Given the ordinal Likert‐scale nature of the data and the clinical sample, which is prone to skewness from response biases like ceiling effects, anticipated non‐normality was addressed through robust CFA estimation. This involved using maximum likelihood with bootstrapping (1000 resamples) to produce bias‐corrected confidence intervals and standard errors, thereby ensuring reliable parameter estimates and fit indices [31, 32]. This approach aligns with COSMIN recommendations for handling distributional assumptions in psychometric validation [33].

The appropriateness of the sample for EFA was evaluated using Bartlett's Test of Sphericity and the Kaiser‐Meyer‐Olkin (KMO) test. The KMO index was categorized as follows: 0.50–0.70 (moderate), 0.70–0.80 (good), 0.80–0.90 (very good), and above 0.90 (excellent). A KMO score above 0.50, along with a significant Bartlett's test, confirmed the adequacy of the sample size for EFA. Latent factors were identified using Principal Axis Factoring with Varimax rotation, and the scree plot was consulted to determine factor retention. Eigenvalues were calculated by solving the correlation matrix's characteristic equation, with each eigenvalue indicating the variance explained by a specific factor. Factors with eigenvalues exceeding one were considered significant, following Kaiser's criterion. Moreover, the scree plot was examined to visually confirm the number of factors by identifying where the curve leveled off [34].

In the second phase, CFA was performed to evaluate the structural model's fit and the model's goodness‐of‐fit indices. This ensured that the obtained data corresponded with the proposed factor structure in the original scale. Data from all participants (339 individuals) were utilized to maintain the desired sample size ratio to the number of observed variables (at least 5 to 10 times the number of items in the scale). Following the recommendations of Jaccard and Wan, as well as Meyers et al. [35], five indices were examined to determine model fit: the chi‐square/degrees of freedom ratio (CMIN/DF), Goodness‐of‐Fit Index (GFI), Adjusted Goodness‐of‐Fit Index (AGFI), Root Mean Square Error of Approximation (RMSEA), and Comparative Fit Index (CFI). The threshold for the fit of these indices is presented in Table 5 [20].

Standardized Factor Loadings greater than 0.40 were considered acceptable, indicating strong relationships between questions and scale dimensions. If necessary, the model could be modified using Modification Indicators (MI). To evaluate the convergent validity of the SACS scale, the Fornell and Larcker approach was utilized [36], incorporating Average Variance Extracted (AVE) and Composite Reliability (CR) as key metrics. For establishing convergent validity, it is suggested that AVE values surpass 0.5, while CR values should exceed 0.7, ensuring the scale's items reliably measure the intended constructs. All analyses were conducted using SPSS‐AMOS24 software. Notably, no missing data were observed in this study, as all participants fully completed the questionnaire. This was achieved through rigorous inclusion/exclusion criteria and meticulous data collection oversight.

##### 2.2.2.4 Convergent Validity

One standard method for examining a scale's construct validity is convergent validity. In this approach, the researcher compares the correlation between the data obtained from the scale under investigation and a scale validated in previous studies [21]. This study utilized the Persian version of the patient self‐advocacy scale (PSAS‐P) to assess convergent validity.

##### 2.2.2.5 Reliability

In this study, reliability was assessed using two methods: internal consistency and stability. The instrument's internal consistency was determined using both Cronbach's alpha (α) and McDonald's omega (ω) for the entire scale and each factor. Values of α and ω greater than 0.7 were considered acceptable [37]. Given that self‐advocacy is a variable likely to exhibit minimal changes over a short period, the stability of the scale was evaluated using the test–retest method. In this approach, 30 participants completed the questionnaire once and then responded to the questions again 2 weeks later. The ICC was calculated; an acceptable ICC value in this study was considered to be 0.75 or higher [38].

## 3 Results

### 3.1 Participant Characteristics

This study's total number of participants was 339, with an average age of 57.18 ± 13.87 years. Among the participants, 51.60% were women and 48.40% were men. Most individuals involved in the research were married (79.60%). Regarding education, most patients were either illiterate (38.60%) or had a low level of education (38.10%). Based on their current employment status, participants were categorized into groups: unemployed (75.20%), part‐time employed (4.70%), full‐time employed (7.70%), and retired (12.40%). Furthermore, approximately 85% of the patients reported having health insurance. Regarding cancer types, 24.20% of the patients had stomach cancer (82 individuals), 17.40% had breast cancer (59 individuals), and 17.10% had colorectal cancer (58 individuals). It is important to note that since all participating patients were oncology clinic attendees receiving anti‐cancer medications, this detail was omitted from the demographic information. Additional demographic information about the participating patients is provided in detail in Table 1.

**TABLE 1:** Demographic characteristics of participants.

Demographic characteristics	Frequency (percentage)
Gender	
Female	175 (51.60)
Male	164 (48.40)
Marital status	
Single	21 (6.20)
Married	270 (79.60)
Widow	48 (14.20)
Educational status	
Illiterate	131 (38.60)
Educated under diploma	129 (38.10)
Diploma	51 (15.00)
Bachelor's degree	21 (6.20)
Master's degree and above	7 (2.10)
Employment status	
Unemployed	255 (75.20)
Part‐time	16 (4.70)
Full‐time	26 (7.70)
Retired	42 (12.40)
Annual family income	
Less than 50 million Tomans	82 (24.20)
Between 50 and 100 million Tomans	88 (26.00)
Between 100 and 150 million Tomans	75 (22.10)
More than 150 million Tomans	43 (12.70)
No answer	51 (15.00)
Type of insurance	
With insurance	287 (84.60)
Supplementary insurance	42 (12.40)
Without insurance	10 (2.90)
Income‐cost matching	
Yes	164 (48.40)
No	175 (51.60)
Type of cancer	
Head and neck	5 (1.50)
Lymphoma	26 (7.70)
Multiple myeloma	9 (2.70)
Esophagus	4 (1.20)
Lung	20 (5.90)
Colorectal	58 (17.10)
Leukemia	22 (6.50)
Breast	59 (17.40)
Stomach	82 (24.20)
Ovarian	16 (4.70)
Other	38 (11.10)
Age (mean ± SD)	57.18 ± 13.87
Time elapsed since diagnosis	16.40 ± 22.05
Total participants	339 (100.00)

### 3.2 Face Validity

The scores derived from the item impact formula for the items in the scale varied between 2.28 and 5, with all items obtaining scores exceeding 1.5 in this assessment. This suggests that the items were adequately comprehensible to the target population. Consequently, all items were considered suitable, and none were eliminated. Nevertheless, to enhance the comprehensibility of the items, a qualitative evaluation of face validity was performed, leading to minor adjustments based on patients' feedback. During this process, several patients indicated that the repeated use of the term “cancer” in the questionnaire made them feel uneasy. As a result, after discussions with the research team, the term “cancer” was substituted with “disease” in several questions (though not all) while preserving the intended meaning. Additionally, two patients pointed out that questions 11 and 20 were redundant, as both pertained to trust in healthcare providers.

### 3.3 Content Validity

The obtained CVR ranged from 0.2 to 1. Notably, items 4, 8, 17, 20, and 29 received CVR values below 0.62, leading specialists to classify them as unnecessary, such as cultural irrelevance or redundancy, according to Lawshe's table [29].

Additionally, the CVI values varied from 0.6 to 1. Items 4, 8, 17, and 20, which scored below 0.79, were deemed inappropriate. Consequently, due to their scores falling below the acceptable thresholds for CVR and CVI, these items were removed from the Persian version of the questionnaire. The omitted items are marked with an asterisk in Table 2, which lists all the original scale items. Overall, the content validity was supported by a high scale‐level CVI (S‐CVI) of 0.91.

**TABLE 2:** Original SACS scale items with removed items in the Persian version marked with an asterisk.

Items	The scale items
1	I have the knowledge to solve the problems I face as a cancer patient.
2	I seek out information before making decisions about my cancer care.
3	I weigh my options carefully before making important decisions about my cancer care.
4	I know what to expect with my cancer.*
5	I feel lost when I have to make decisions about my cancer care.
6	I feel lost when I don't know what my treatment plan is.
7	I can express my priorities to my provider when making decisions about my cancer care.
8	I am comfortable asking for a second opinion.*
9	I search for the top provider to treat my cancer.
10	I know where to get an answer if my provider doesn't have one.
11	I trust the plan my provider has made for me.
12	I ask questions when I don't understand what my provider is telling me.
13	I talk to my provider if I don't agree with his or her recommendations.
14	My job is to follow my provider's recommendations.
15	I feel uncomfortable raising concerns about my care to my provider.
16	I rarely tell my provider about the problems I am having.
17	I tell my provider about problems that are unexpected.*
18	I have a hard time voicing my preferences to my provider.
19	I ask my provider to explain his or her recommendations.
20	I rely on my provider's skills to manage my cancer.*
21	I seek out support from other cancer patients.
22	Helping other cancer patients also helps me.
23	When I hear someone has cancer, I try to reach out to them.
24	I reach out to someone with cancer if it will help them.
25	It helps me to know that other cancer patients have gone through what I am going through.
26	Telling others how I'm doing makes me feel better.
27	I know who to ask to get support.
28	I try to raise awareness about cancer.
29	I am careful with who I tell about my cancer.*

* Omitted items.

### 3.4 Construct Validity

#### 3.4.1 Normality Assessments

The normality assessments confirmed expected deviations consistent with ordinal data in a sample of cancer patients. The K‐S test on the total score indicated significant non‐normality (*D* = 0.078, *p* < 0.001, Lilliefors correction). Item‐level univariate analyses revealed substantial skewness (range: −1.320 to 0.837; mean = −0.778; c.r. range: −10.984 to 8.631, with |c.r.| > 1.96 for 22 out of 24 items) and kurtosis (range: −1.069 to 3.408; mean = 1.12; c.r. range: −5.554 to 12.809, significant for most items). Multivariate evaluation using Mardia's coefficient further supported non‐normality (skewness β₁ = −0.778, c.r. = −5.850; kurtosis β₂ = 210.604, c.r. = 54.882; both *p* < 0.001). Despite these violations, bootstrapped CFA results demonstrated model robustness, with fit indices remaining within acceptable thresholds (see Table 4). This confirms the stability of the factor structure and supports the scale's construct validity in the Persian context.

#### 3.4.2 EFA

In this study, the KMO was 0.90, and Bartlett's test yielded a value of 4316.16 (df = 276), *p* < 0.001. Principal Axis Factoring (PAF) was utilized to extract latent factors due to its effectiveness in psychometric scale validation. PAF focuses on common variances and minimizes the influence of unique and error variance. This approach aligns with the study's objective to reveal the underlying factor structure of the Self‐Advocacy in Cancer Survivorship Scale (SACS‐P) [39].

To ensure the appropriateness of the EFA, the study assessed the univariate and multivariate distributions of the data. Univariate normality was examined for each item of the SACS scale by calculating skewness and kurtosis, with values within the range of ±2 indicating acceptable normality. Multivariate normality was not formally tested, as Principal Axis Factoring is robust to moderate violations of this assumption. This robustness justified the use of PAF, ensuring reliable factor extraction despite potential deviations from multivariate normality.

By using PAF combined with varimax rotation and scree plot analysis, three factors with eigenvalues exceeding one were identified. These factors had eigenvalues of 4.76, 4.30, and 3.62, respectively, together explaining 52.85% of the total variance in the scale (Table 3). This method ensured the extraction of orthogonal factors and facilitated clear interpretation of the factor structure.

**TABLE 3:** Extracted factors in EFA.

Latent factor	Item	Factor 1	Factor 2	Factor 3	Communalities	Eigenvalue	% of variance
Informed decision‐making	1	0.76			0.62	4.76	19.84
	2	0.80			0.66		
	3	0.75			0.59		
	4	0.59			0.39		
	5	0.70			0.52		
	6	0.73			0.58		
	7	0.71			0.54		
	8	0.59			0.40		
	9	0.63			0.41		
Effective communication with healthcare providers	10		0.73		0.56	4.30	17.92
	11		0.68		0.51		
	12		0.60		0.37		
	13		0.66		0.46		
	14		0.75		0.59		
	15		0.70		0.54		
	16		0.70		0.53		
Connected strength	17			0.73	0.56	3.62	15.84
	18			0.87	0.76		
	19			0.73	0.54		
	20			0.79	0.64		
	21			0.63	0.40		
	22			0.77	0.60		
	23			0.63	0.43		
	24			0.54	0.37		
Total (SACS‐P)						12.68	52.85

#### 3.4.3 CFA

**FIGURE 1:**
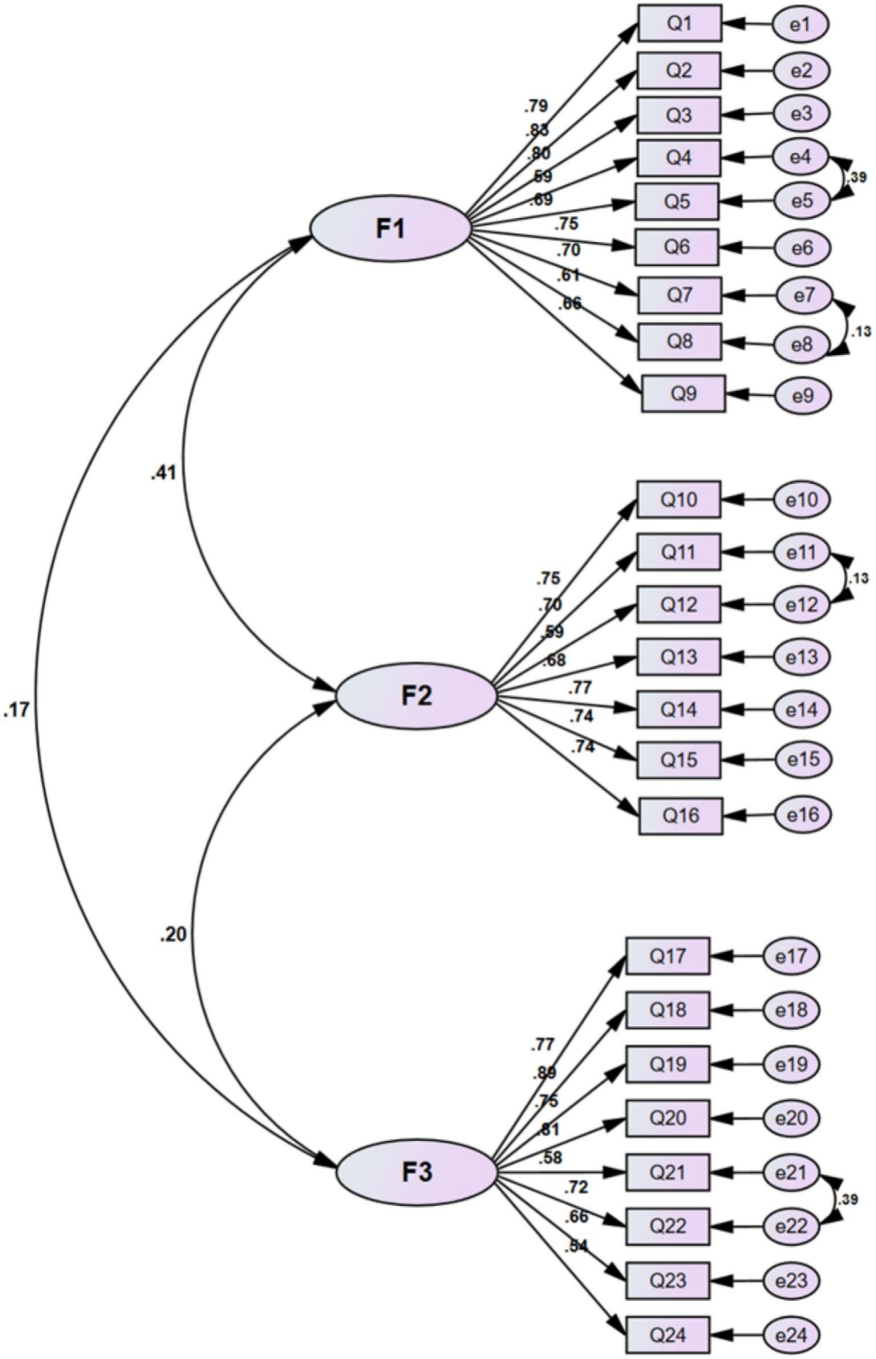
The confirmatory factor analysis model of the Persian version of the SACS (*n* = 339). F1, Informed decision‐making; F2, effective communication with healthcare providers; F3, connected strength.

Figure 1 illustrates the structural model of the Persian version of the SACS scale, which consists of 24 items across three factors. F1, F2, and F3 in Figure 1 represent the factors of informed decision‐making (with nine items), effective communication with healthcare providers (with seven items), and connected strength (with eight items), respectively. The factor loadings for all items in the scale were above 0.3; therefore, no items were removed at this stage (*p* < 0.001).

Additionally, several indices were evaluated to assess the goodness of fit for the 24‐item factor structure (see Table 4). All indices, except for the NFI, indicated an adequate fit for the final model. Based on the Modification Indices, modifications were made to the final model; specifically, correlations were identified between items 4 and 5 (e4, e5), 7 and 8 (e7, e8), 11 and 12 (e11, e12), and 21 and 22 (e21, e22).

**TABLE 4:** Indicators of the Persian version of the SACS scale (24 items).

Indicator	Model estimates	Permissible limit	Result
*χ* ^2^/df	1.86	≤ 3	Approved
NFI	0.89	≥ 0.90	Not approved
CFI	0.94	≥ 0.90	Approved
TLI	0.94	≥ 0.90	Approved
IFI	0.95	≥ 0.90	Approved
GFI	0.9	≥ 0.90	Approved
PNFI	0.79	≥ 0.50	Approved
PCFI	0.84	≥ 0.50	Approved
SRMR	0	≤ 0.10	Approved
RMSEA	May‐00	≤ 0.08	Approved

Abbreviations: CFI, comparative fit index; GFI, goodness of fit index; IFI, incremental fit index; NFI, normed fit index; PCFI, parsimony comparative fit index; PNFI, parsimonious normed fit index; RMSEA, root mean square error of approximation; SRMR, standardized root mean square residual; TLI, Tucker‐Lewis index; *χ* ^2^/df, Chi‐square to degrees of freedom ratio.

### 3.5 Convergent and Discriminant Validity, Construct Reliability

Convergent and discriminant validity, along with construct reliability, were evaluated in this study based on the Fornell & Larcker approach. The composite reliability (CR) and average variance extracted (AVE) values exceeded acceptable thresholds (see Table 5), indicating robust convergent validity. The results confirmed that MSV was consistently lower than AVE, as reported in the analysis, suggesting strong discriminant validity.

**TABLE 5:** Convergent and divergent validity indices of the Persian version of the SACS scale.

Factor	CR	AVE	MSV
Informed decision‐making	0.90	0.51	0.16
Effective communication with healthcare providers	0.78	0.51	0.16
Connected strength	0.89	0.52	0.04

Abbreviations: AVE, average variance extracted; CR, composite reliability; MSV, maximum shared squared variance.

### 3.6 Convergent Validity

This section evaluated the relationship between the data collected from the SACS‐P and PSAS‐P scales using the Spearman correlation test, as the data did not follow a normal distribution. The correlation coefficient was *r* = 0.49 (*p* < 0.001). This result suggests that the two instruments are statistically associated and demonstrate a moderate level of correlation. Considering that, in the context of convergent validity, the correlation between two instruments should ideally not be excessively high, with moderately strong correlations being favored [20] it can be concluded that the SACS‐P scale demonstrates a suitable level of convergent validity. Nonetheless, while both instruments assess the same construct, they possess differences that may account for the moderate correlation observed between them. For instance, the SACS scale targets cancer patients who experience distinct and specific physical, psychological, and social challenges compared to those with other chronic conditions. Furthermore, the SACS scale encompasses a more significant number of items and dimensions.

### 3.7 Reliability

The findings indicate that the scale's internal consistency was evaluated through two coefficients: Cronbach's alpha and omega. The Cronbach's alpha values for each factor of the scale and the entire scale ranged from 0.87 to 0.90. Furthermore, the omega coefficient yielded comparable results, which support the instrument's reliability. The scale's stability was assessed using the test–retest method, revealing ICC values of 0.77, 0.79, and 0.76 for the different dimensions and an overall value of 0.75 for the entire instrument (Table 6).

**TABLE 6:** Cronbach's alpha coefficient for the three‐factor model, 24 items (*n* = 339).

Factor	Number of items	Cronbach's alpha	Omega	ICC
Informed decision‐making	9	0.90	0.90	0.77
Effective communication with healthcare providers	7	0.87	0.87	0.79
Connected strength	8	0.89	0.88	0.76
Total	24	0.89	0.88	0.75

## 4 Discussion

This study assessed the psychometric properties of the Persian version of the Self‐Advocacy in Cancer Survivorship Scale (SACS‐P) among Iranian cancer patients, showing acceptable validity and reliability. The results indicate that the SACS‐P is a valuable scale for evaluating self‐advocacy in this group, with potential benefits for improving patient‐centered care in Iran. These insights help to enhance our comprehension of self‐advocacy in this at‐risk population.

A primary strength of this study is its rigorous adherence to the COSMIN checklist, ensuring a comprehensive evaluation of the SACS‐P's psychometric properties, including content and face validity, construct validity, internal consistency, and stability. The inclusion of a diverse sample of cancer patients enhances the generalizability of the findings, capturing a broad range of experiences within the Iranian context [40]. The demographic profile of participants, predominantly married and unemployed, underscores the profound socioeconomic impact of cancer, which often disrupts familial and professional responsibilities [41]. This context amplifies the importance of self‐advocacy, enabling patients to navigate a complex healthcare system, access limited resources, and address their medical and psychosocial needs effectively. Ensuring the relevance and comprehensiveness of the scale's items was critical to establishing content validity and tailoring the instrument to these challenges.

The process of establishing face and content validity revealed critical insights into the cultural relevance of the SACS‐P. While initial item impact scores suggested adequate comprehensibility, qualitative feedback highlighted the need for modifications to enhance patient comfort and understanding. The seemingly minor substitution of “cancer” with “disease” in specific questions underscores the sensitivity surrounding cancer terminology and the potential for such language to evoke unease among patients. This adjustment reflects a crucial aspect of cultural adaptation, ensuring that the instrument resonates with the target population's lived experiences and emotional sensitivities. In line with this, it is noteworthy that items identified by specialists as lacking necessity or relevance to the measured construct, which received scores below the established threshold, were eliminated to improve the scale's validity.

Thomas et al. also evaluated face validity during the development of the FSACS scale, which subsequently informed the creation of the SACS scale [17]. However, their publication did not document the impact scores for the 57 scale items. While their study did not report impact scores for the scale items, this study confirmed that all items exceeded the established threshold, with minor adjustments made based on patient feedback. Thomas et al. also assessed the content validity of the FSACS scale, removing several items based on their results; unlike the original study, which did not provide content validity index (CVI) or content validity ratio (CVR) values, this study's comprehensive assessment, combining quantitative and qualitative methods, ensured a robust evaluation of the SACS‐P's cultural and contextual relevance [42].

Exploratory factor analysis (EFA) revealed a three‐factor structure—informed decision‐making, effective communication with healthcare providers, and connected strength—consistent with the original SACS scale [18]. This consistency suggests that these core dimensions of self‐advocacy are relevant across cultural contexts. CFA further supported the goodness of fit for the factor structure, with most indices indicating an adequate model fit. Correlations between certain items, identified through Modification Indices, suggest potential overlap or shared variance, particularly in aspects of informed decision‐making and effective communication. These findings reflect the interconnected nature of self‐advocacy components in the experiences of Iranian cancer patients. The study also provided strong evidence for convergent validity [18], with a moderate correlation between the SACS‐P and the Persian version of the Patient Self‐Advocacy Scale (PSAS‐P) [19]. This correlation indicates that while the scales share some conceptual overlap, the SACS‐P uniquely addresses the specific challenges faced by cancer patients, unlike the PSAS‐P, which is designed for broader chronic disease populations. A moderate correlation is desirable, as an overly strong correlation would suggest redundancy, undermining the need for a cancer‐specific instrument [20].

Comparative studies, such as the validation of the FSACS among women with cancer in China, reported similar findings, including a consistent three‐factor structure and strong content validity [43]. However, unlike the current study, the Chinese study did not remove items during content validity evaluation and assessed criterion validity rather than convergent validity, likely due to the absence of a comparable instrument in that context. The high internal consistency across all factors and the entire SACS‐P scale confirm that the items reliably measure the same underlying constructs. Test–retest reliability further supports the scale's stability over time, ensuring its utility for longitudinal assessments.

## 5 Conclusion

The Persian version of the Self‐Advocacy in Cancer Survivorship Scale (SACS‐P) showed strong psychometric properties in Iranian cancer patients. After removing culturally inappropriate or redundant items, it demonstrated high face and content validity. Construct validity was confirmed through exploratory and confirmatory factor analyses, identifying three factors explaining significant variance with good model fit. The scale exhibited strong convergent and discriminant validity, excellent internal consistency (Cronbach's alpha and omega above thresholds), and temporal stability via test–retest reliability. The SACS‐P is a reliable and valid instrument for assessing self‐advocacy across diverse Iranian cancer patients, supporting its use in identifying needs and promoting engagement in care to improve health outcomes in cancer survivorship.

## 6 Limitations

This study acknowledges several limitations. Firstly, as a self‐report measure, the SACS scale may be susceptible to biases such as socially desirable responding or vague self‐assessment, potentially impacting the accuracy of participants' reports. Additionally, voluntary participation could exclude individuals with lower self‐advocacy skills, who may struggle to express their needs, thus potentially biasing the sample. The sample was restricted to patients attending oncology clinics and receiving active anti‐cancer treatment, limiting the generalizability to the broader Iranian cancer population, especially those not currently undergoing treatment. Furthermore, the cross‐sectional design restricts the ability to draw causal conclusions regarding self‐advocacy and related variables. Another limitation was patients' discomfort with the term “cancer,” which may have hindered responses; this was addressed by substituting “disease” where appropriate after consulting with the instrument's designer. Lastly, the relatively low literacy level among many participants posed challenges in assessing face validity, which was managed by involving patients with average literacy in this phase. Future studies should aim to utilize more diverse samples, longitudinal designs, and explore interventions to enhance self‐advocacy and its impact on psychological outcomes.

## 7 Implications (Clinical and Research)

The Persian version of the Self‐Advocacy in Cancer Survivorship Scale (SACS‐P) is a reliable scale for evaluating self‐advocacy in Iranian cancer patients. It has strong validity, internal consistency, and temporal stability, measuring three key factors: informed decision‐making, effective communication with healthcare providers, and connected strength. The SACS‐P can help healthcare professionals identify patient needs and create targeted interventions for better care engagement. It is suitable for diverse Iranian populations with different demographic characteristics, and its use can enhance patient‐provider communication and health outcomes. Future research could explore its application in longitudinal studies or other cultural contexts.

## Author Contributions

Conceptualization: E.H. and M.S. Data curation: E.H., N.R., M.S. Formal analysis: E.H., S.G., M.S., N.R. Investigation: E.H., N.R. Methodology: S.G., N.R., M.S., E.H. Supervision: M.S. Validation: E.H., S.G., M.S., N.R. Visualization: E.H. Writing – original draft: E.H., N.R., S.G., M.S. Writing – review and editing: M.S., E.H., N.R., S.G.

## Ethics Statement

The study, conducted as part of a master's thesis, received ethical approval from the Ardabil University of Medical Sciences Ethics Board (IR.ARUMS.REC.1403.227, October 7, 2024). Permission for translation and psychometric evaluation of the SACS was obtained from the original author. Eligible participants were selected, and the study's objectives and methods were explained to them. Participants' questions were answered, and they were informed that participation was voluntary and could be withdrawn at any time. Data were collected and managed anonymously and confidentially, with no use beyond the study's purpose. Written informed consent was obtained from all participants. The study adhered to the ethical principles of the Declaration of Helsinki, ensuring participants' rights, safety, and well‐being.

## Conflicts of Interest

The authors declare no conflicts of interest.

## Data Availability

The data that support the findings of this study are available on request from the corresponding author. The data are not publicly available due to privacy or ethical restrictions.

## References

[1] R. L. Siegel , K. D. Miller , H. E. Fuchs , and A. Jemal , “Cancer Statistics, 2022,” CA: A Cancer Journal for Clinicians 72, no. 1 (2022): 7–33, 10.3322/caac.21708. 35020204

[2] H. Sung , J. Ferlay , R. L. Siegel , et al., “Global Cancer Statistics 2020: GLOBOCAN Estimates of Incidence and Mortality Worldwide for 36 Cancers in 185 Countries,” CA: A Cancer Journal for Clinicians 71, no. 3 (2021): 209–249, 10.3322/caac.21660. 33538338

[3] G. Roshandel , J. Ferlay , A. Ghanbari‐Motlagh , et al., “Cancer in Iran 2008 to 2025: Recent Incidence Trends and Short‐Term Predictions of the Future Burden,” International Journal of Cancer 149, no. 3 (2021): 594–605, 10.1002/ijc.33574. 33884608

[4] mediainquirieswho.int , “Global Cancer Burden Growing, Amidst Mounting Need for Services,” accessed April 9, 2025.

[5] M. Jefford , “Improving the Care of Adult Cancer Survivors,” Asia‐Pacific Journal of Oncology Nursing 7, no. 1 (2020): 2–5, 10.4103/apjon.apjon_42_19. 31879677 PMC6927155

[6] V. S. Blinder and F. M. Gany , “Impact of Cancer on Employment,” Journal of Clinical Oncology 38, no. 4 (2020): 302–309, 10.1200/jco.19.01856. 31804857 PMC6992498

[7] S. Ghoshal , M. Arora , A. Chakrabarti , A. Datta , and T. Dey , “The Socio‐Economic Burden of Cancer: An Observation From the Palliative Care OPD,” Journal of Family Medicine and Primary Care 11, no. 3 (2022): 821–824, 10.4103/jfmpc.jfmpc_1247_21. PMC9051693 35495789

[8] M. Schlander , W. van Harten , V. P. Retèl , et al., “The Socioeconomic Impact of Cancer on Patients and Their Relatives: Organisation of European Cancer Institutes Task Force Consensus Recommendations on Conceptual Framework, Taxonomy, and Research Directions,” Lancet Oncology 25, no. 4 (2024): e152–e163, 10.1016/S1470-2045(23)00636-8. 38547899

[9] L. A. Levit , E. Balogh , S. J. Nass , and P. Ganz , Delivering High‐Quality Cancer Care: Charting a New Course for a System in Crisis (National Academies Press, 2013). 24872984

[10] T. L. Hagan , S. Cohen , C. Stone , and H. Donovan , “Theoretical to Tangible: Creating a Measure of Self‐Advocacy for Female Cancer Survivors,” Journal of Nursing Measurement 24, no. 3 (2016): 428–441, 10.1891/1061-3749.24.3.428. 28714448 PMC5514617

[11] T. H. Thomas , H. S. Donovan , M. Q. Rosenzweig , C. M. Bender , and Y. Schenker , “A Conceptual Framework of Self‐Advocacy in Women With Cancer,” Advances in Nursing Science 44, no. 1 (2021): E1–e13, 10.1097/ans.0000000000000342. 33181568 PMC7894983

[12] G. Petri , J. Beadle‐Brown , and J. Bradshaw , “Redefining Self‐Advocacy: A Practice Theory‐Based Approach,” Journal of Policy and Practice in Intellectual Disabilities 17, no. 3 (2020): 207–218, 10.1111/jppi.12343.

[13] T. L. Hagan , S. Gilbertson‐White , S. M. Cohen , J. S. Temel , J. A. Greer , and H. S. Donovan , “Exploring the Relationships Between Patient Self‐Advocacy and Cancer Symptom Burden,” Clinical Journal of Oncology Nursing 22, no. 1 (2018): E23, 10.1188/18.CJON.E23-E30. 29350706 PMC5841467

[14] D. E. Brashers , S. M. Haas , and J. L. Neidig , “The Patient Self‐Advocacy Scale: Measuring Patient Involvement in Health Care Decision‐Making Interactions,” Health Communication 11, no. 2 (1999): 97–121, 10.1207/s15327027hc1102_1. 16370972

[15] J. A. Jonikas , D. D. Grey , M. E. Copeland , et al., “Improving Propensity for Patient Self‐Advocacy Through Wellness Recovery Action Planning: Results of a Randomized Controlled Trial,” Community Mental Health Journal 49 (2013): 260–269, 10.1007/s10597-011-9475-9. 22167660

[16] D. W. Test , C. H. Fowler , W. M. Wood , D. M. Brewer , and S. Eddy , “A Conceptual Framework of Self‐Advocacy for Students With Disabilities,” Remedial and Special Education 26, no. 1 (2005): 43–54, 10.1177/0741932505026001060.

[17] T. L. Hagan , S. M. Cohen , M. Q. Rosenzweig , K. Zorn , C. A. Stone , and H. S. Donovan , “The Female Self‐Advocacy in Cancer Survivorship Scale: A Validation Study,” Journal of Advanced Nursing 74, no. 4 (2018): 976–987, 10.1111/jan.13498. 29117439 PMC5844819

[18] T. H. Thomas , P. W. Scott , M. L. Nilsen , et al., “The Female Self‐Advocacy in Cancer Survivorship Scale Is a Psychometrically Sound Measure of Self‐Advocacy in Male Cancer Survivors,” Psycho‐Oncology 33, no. 1 (2024): e6269, 10.1002/pon.6269. 38095337 PMC10872533

[19] S. Vahdat , L. Hamzehgardeshi , Z. Hamzehgardeshi , and S. Hessam , “Psychometric Properties of the Patient Self‐Advocacy Scale: The Persian Version,” Iranian Journal of Medical Sciences 40, no. 4 (2015): 349–355. 26170522 PMC4487461

[20] D. L. Streiner , G. R. Norman , and J. Cairney , Health Measurement Scales: A Practical Guide to Their Development and Use (Oxford University Press, 2024), 10.1093/med/9780192869487.001.0001.

[21] A. C. Curtis and C. Keeler , “Measurement in Nursing Research,” American Journal of Nursing 121, no. 6 (2021): 56–60, 10.1097/01.NAJ.0000753668.78872.0f. 34009166

[22] S.‐C. Chow , J. Shao , H. Wang , and Y. Lokhnygina , Sample Size Calculations in Clinical Research (Chapman and Hall/CRC, 2017), https://lccn.loc.gov/2017011239.

[23] World Health Organization , Global Scales for Early Development v1 0: Adaptation and Translation Guide (World Health Organization, 2023), https://www.who.int/publications/i/item/WHO‐MSD‐GSED‐package‐v1.0‐2023.1.

[24] D. Polit and C. Beck , Essentials of Nursing Research: Appraising Evidence for Nursing Practice, 9th ed. (Wolters Kluwer, 2018).

[25] C. Van Der Vleuten and L. Bouter , “COSMIN Risk of Bias Tool to Assess the Quality of Studies on Reliability or Measurement Error of Outcome Measurement Instruments: A Delphi Study,” BMC Medical Research Methodology 20 (2020): 1–13, 10.1186/s12874-020-01179-5. PMC7712525 33267819

[26] D. Polit‐O'Hara and F. M. Yang , “Measurement and the Measurement of Change: A Primer for the Health Professions,” (2016).

[27] B. Nevo , “Face Validity Revisited,” Journal of Educational Measurement 22, no. 4 (1985): 287–293, 10.1111/j.1745-3984.1985.tb01065.x.

[28] M. S. Allen , D. A. Robson , and D. Iliescu , “Face Validity,” European Journal of Psychological Assessment 39 (2023): 153–156, 10.1027/1015-5759/a000777.

[29] C. H. Lawshe , “A Quantitative Approach to Content Validity,” Personnel Psychology 28, no. 4 (1975): 563–575.

[30] E. Almanasreh , R. Moles , and T. F. Chen , “Evaluation of Methods Used for Estimating Content Validity,” Research in Social and Administrative Pharmacy 15, no. 2 (2019): 214–221, 10.1016/j.sapharm.2018.03.066. 29606610

[31] B. M. Byrne , Structural Equation Modeling With Mplus: Basic Concepts, Applications, and Programming (Routledge, 2013), 10.4324/9780203807644.

[32] K. A. Markus , “Review of Principles and Practice of Structural Equation Modeling,” (2012), 10.1080/10705511.2012.687667.

[33] L. B. Mokkink , H. C. De Vet , C. A. Prinsen , et al., “COSMIN Risk of Bias Checklist for Systematic Reviews of Patient‐Reported Outcome Measures,” Quality of Life Research 27, no. 5 (2018): 1171–1179, 10.1007/s11136-017-1765-4. 29260445 PMC5891552

[34] N. Shrestha , “Factor Analysis as a Tool for Survey Analysis,” American Journal of Applied Mathematics and Statistics 9, no. 1 (2021): 4–11, 10.12691/ajams-9-1-2.

[35] L. S. Meyers , G. Gamst , and A. J. Guarino , Applied Multivariate Research: Design and Interpretation (Sage publications, 2016).

[36] C. Fornell and D. F. Larcker , “Evaluating Structural Equation Models With Unobservable Variables and Measurement Error,” Journal of Marketing Research 18, no. 1 (1981): 39–50, 10.1177/002224378101800104.

[37] M. T. Kalkbrenner , “Alpha, Omega, and H Internal Consistency Reliability Estimates: Reviewing These Options and When to Use Them,” Counseling Outcome Research and Evaluation 14, no. 1 (2023): 77–88, 10.1080/21501378.2021.1940118.

[38] C. Xue , J. Yuan , G. G. Lo , et al., “Radiomics Feature Reliability Assessed by Intraclass Correlation Coefficient: A Systematic Review,” Quantitative Imaging in Medicine and Surgery 11, no. 10 (2021): 4431–4460, 10.21037/qims-21-86. 34603997 PMC8408801

[39] A. B. Costello and J. Osborne , “Best Practices in Exploratory Factor Analysis: Four Recommendations for Getting the Most From Your Analysis,” Practical Assessment, Research and Evaluation 10, no. 1 (2005): 1–9, 10.7275/jyj1-4868.

[40] M. Al Maqbali , J. Gracey , J. Rankin , L. Dunwoody , E. Hacker , and C. Hughes , “Cross‐Cultural Adaptation and Psychometric Properties of Quality of Life Scales for Arabic‐Speaking Adults: A Systematic Review,” Sultan Qaboos University Medical Journal 20, no. 2 (2020): e125–e137, 10.18295/squmj.2020.20.02.002. 32655904 PMC7328836

[41] L. Haghjou , L. Mounesan , A. Shamsi , et al., “The Impact of Economic Sanctions on Cancer Diagnosis and Treatment in Iran: A Qualitative Study,” International Journal for Equity in Health 23, no. 1 (2024): 258, 10.1186/s12939-024-02335-9. 39616398 PMC11607833

[42] K. Hyrkäs , K. Appelqvist‐Schmidlechner , and L. Oksa , “Validating an Instrument for Clinical Supervision Using an Expert Panel,” International Journal of Nursing Studies 40, no. 6 (2003): 619–625, 10.1016/S0020-7489(03)00036-1. 12834927

[43] M. Deng , Z. Lu , A. Wang , et al., “Psychometric Properties of the Chinese Version of Female Self‐Advocacy in Cancer Survivorship Scale,” Asia‐Pacific Journal of Oncology Nursing 9, no. 9 (2022): 100080, 10.1016/j.apjon.2022.100080. 36060834 PMC9429498

